# Centimeter Scale Patterned Growth of Vertically Stacked Few Layer Only 2D MoS_2_/WS_2_ van der Waals Heterostructure

**DOI:** 10.1038/srep25456

**Published:** 2016-05-05

**Authors:** Nitin Choudhary, Juhong Park, Jun Yeon Hwang, Hee-Suk Chung, Kenneth H. Dumas, Saiful I. Khondaker, Wonbong Choi, Yeonwoong Jung

**Affiliations:** 1 Nanoscience Technology Center, University of Central Florida, Orlando, Florida 32826, United States; 2 Department of Materials Science and Engineering, University of North Texas, Denton, Texas 76207, United States; 3 Carbon Convergence Materials Research Center, Korea Institute of Science and Technology, Jeonbuk, 565-905, South Korea; 4 Jeonju Center, Korea Basic Science Institute, Jeonju, Jeollabuk-do, 54907, South Korea; 5 Department of Physics, University of Central Florida, Orlando, Florida 32826, United States; 6 Department of Materials Science and Engineering, University of Central Florida, Orlando, Florida 32816, United States

## Abstract

Two-dimensional (2D) van der Waal (vdW) heterostructures composed of vertically-stacked multiple transition metal dichalcogenides (TMDs) such as molybdenum disulfide (MoS_2_) and tungsten disulfide (WS_2_) are envisioned to present unprecedented materials properties unobtainable from any other material systems. Conventional fabrications of these hybrid materials have relied on the low-yield manual exfoliation and stacking of individual 2D TMD layers, which remain impractical for scaled-up applications. Attempts to chemically synthesize these materials have been recently pursued, which are presently limited to randomly and scarcely grown 2D layers with uncontrolled layer numbers on very small areas. Here, we report the chemical vapor deposition (CVD) growth of large-area (>2 cm^2^) patterned 2D vdW heterostructures composed of few layer, vertically-stacked MoS_2_ and WS_2_. Detailed structural characterizations by Raman spectroscopy and high-resolution/scanning transmission electron microscopy (HRTEM/STEM) directly evidence the structural integrity of two distinct 2D TMD layers with atomically sharp vdW heterointerfaces. Electrical transport measurements of these materials reveal diode-like behavior with clear current rectification, further confirming the formation of high-quality heterointerfaces. The intrinsic scalability and controllability of the CVD method presented in this study opens up a wide range of opportunities for emerging applications based on the unconventional functionalities of these uniquely structured materials.

The quest for the fundamental physics and exciting new phenomenon inherent to 2D TMDs has opened new avenues in the field of 2D vdW heterostructures^1^,^2^,^3^. Motivated by the well-established heterojunction engineering of traditional semiconductor thin films, developing new hybrid materials by stacking up dissimilar 2D TMDs allows for the realization of unique and superior materials properties that cannot be obtained otherwise^1^,^2^. For example, theoretical^4^,^5^,^6^,^7^,^8^,^9^,^10^ and experimental^11^,^12^,^13^,^14^,^15^,^16^,^17^,^18^,^19^,^20^,^21^ studies have demonstrated intriguing band alignment and tunneling transports as well as fast charge transfer and strong interlayer coupling in vertically-stacked 2D heterostructures employing molybdenum (Mo) or tungsten (W)-based TMDs. An important attribute of these atomically assembled hybrid materials is the seamless stitching of dissimilar 2D TMDs via weak vdW forces benefiting from relaxed lattice mismatch constriction^1^. The anisotropic bonding nature of the layered TMDs also enables them to grow aligning their 2D layers in two distinct directions^22^,^23^,^24^, further emphasizing the importance of controlling their morphology for desired materials functionalities. Thus, establishing reliable methods that can stack up multiple 2D TMDs with well-defined components and orientations will greatly broaden their horizons in a wide range of applications such as flexible electronics and optoelectronics utilizing their extraordinary opto-electrical properties and extremely high strain limit^25^,^26^. Considerable efforts to integrate vertically-stacked 2D TMDs have been mainly driven by the manual exfoliation and stacking of individual 2D layers as demonstrated in MoS_2_/WSe_2_^11^,^12^,^13^,^14^,^15^,^16^, MoS_2_/WS_2_^17^,^18^, MoS_2_/MoSe_2_^19^, and MoSe_2_/WSe_2_^20^. However, this mechanical transfer approach produces 2D layers with low yield and arbitrary spatial distribution, intrinsically lacking a capability of scalable materials production. Alternatively, chemical vapor deposition (CVD) has been employed for the growth of large-area TMDs, and it has recently been extended to grow vertically-stacked 2D TMD heterostructures. A few successful CVD growths of vertically-stacked WS_2_/MoS_2_^21^,^27^,^28^, WSe_2_/MoS_2_ (or, MoS_2_/WSe_2_)^29^, and WSe_2_/MoSe_2_^30^ as well as vdW heteroepitaxy-based MoS_2_ heterostructures^31^ have been demonstrated either via the co-reaction of metal-based precursors with chalcogens or via the sequential growth of one material on the other. However, all these CVD-grown 2D TMD heterostructures are presently limited to be demonstrated on very small areas lacking a control of their location, size, thickness and uniformity. Thus, much effort is still needed to develop viable approaches to synthesize vertically-stacked 2D TMD heterostructures in a highly scalable and controlled manner to realize their true potential.

Herein, we report the scalable and patterned CVD growth of vertically-stacked few layer 2D MoS_2_/WS_2_ heterostructures with well-defined heterointerfaces on a large area (>2 cm^2^). Detailed structural and electrical characterizations reveal that these hybrid materials well preserve their structural integrity and intrinsic electrical properties of individual constituting 2D TMDs.

## Results and Discussion

**Figure 1:**
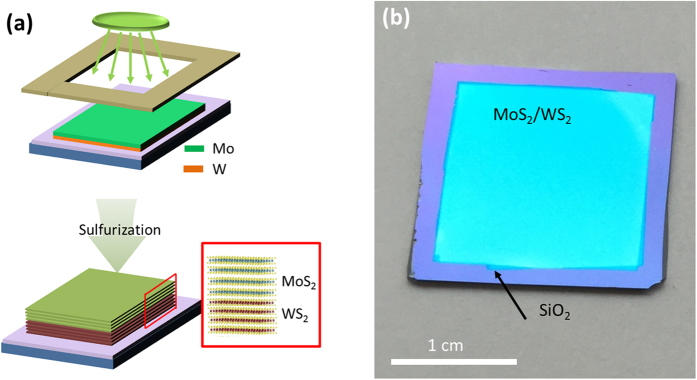
**(a)** Schematic for the large-area, patterned CVD growth of few-layer only, vertically-stacked 2D MoS_2_/WS_2_ heterostructure films. Mo and W films are sequentially patterned and deposited on SiO_2_/Si growth substrates. Subsequent sulfurization converts the Mo and W to few layer 2D MoS_2_ and WS_2,_ respectively. **(b)** Optical image of an as-grown vertically-stacked 2D MoS_2_/WS_2_ heterostructure film on a SiO_2_/Si substrate.

Few layer, vertically-stacked 2D MoS_2_/WS_2_ heterostructures were grown using a two-step process of metal deposition followed by sulfurization in a low-pressure CVD (LPCVD) chamber. Figure 1(a) is a schematic illustration of the growth procedure. Stacks of high quality W and Mo films were sequentially deposited on silicon dioxide (SiO_2_)/silicon (Si) wafers using magnetron sputtering of W and Mo targets, respectively. A metal shadow mask was used for the patterned deposition of Mo/W stacked films. The deposited metal films were subsequently sulfurized in a LPCVD furnace at 600 °C under argon (Ar) environment, which converts Mo and W to MoS_2_ and WS_2_, respectively. As a result, vertically-stacked 2D MoS_2_/WS_2_ films composed of few layer MoS_2_ and WS_2_ were realized only on the areas where Mo and W were initially deposited. Figure 1(b) is an optical image of a vertically-stacked 2D MoS_2_/WS_2_ heterostructure film, demonstrating the scalability and patternability of the presented method.

**Figure 2:**
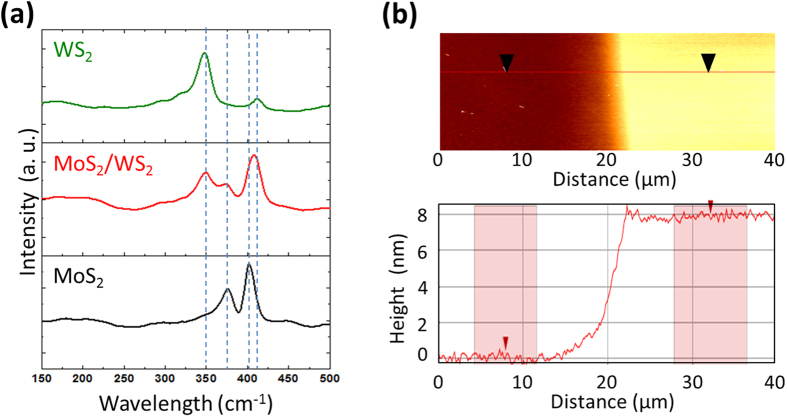
**(a)** Comparison of Raman spectra from WS_2_-only, vertically-stacked MoS_2_/WS_2_ heterostructure, and MoS_2_-only films. **(b)** AFM height profile measurement across a vertically-stacked MoS_2_/WS_2_ heterostructure film.

Raman spectroscopy has become a very powerful tool for studying various 2D materials to quantify their atomic layer numbers^32^. The Raman spectra in Fig. 2(a) confirms the typical in-plane (E′) and out of plane (A′_1_) vibration modes of individual WS_2_ (green) and MoS_2_ (black) films collected at 532 nm laser line. The Raman spectrum from the MoS_2_/WS_2_ heterostructure film (red) exhibits the distinguishable peaks corresponding to a summation of the Raman modes from each WS_2_ and MoS_2_. This observation indicates that our CVD method indeed yields heterostructures that maintain the characteristics of individual constituent 2D TMDs rather than alloyed Mo_x_W_1−x_S_2_ which generally exhibit Raman peaks positioned intermediary to the those from pure MoS_2_ or WS_2_^33^,^34^. The difference between the E′ and A′_1_ modes (Δ*f* ) is a reliable quantity to determine the number of 2D atomic layers^35^. The Δ*f* calculated from the individual WS_2_ and MoS_2_ films were 65 cm^−1^ and 24 cm^−1^, respectively, which correspond to the formation of ~4–5 atomic layers in each material^36^,^37^. Furthermore, atomic force microscopy (AFM) height profile measurements were performed across the edge of an as-grown MoS_2_/WS_2_ heterostructure film to assess its thickness (Fig. 2(b)). The average height of the film as measured from the underlying SiO_2_/Si substrate surface corresponds to ~7–8 nm indicating a growth of ~4–5 layers for each MoS_2_ and WS_2_, consistent with the Raman characterization.

**Figure 3:**
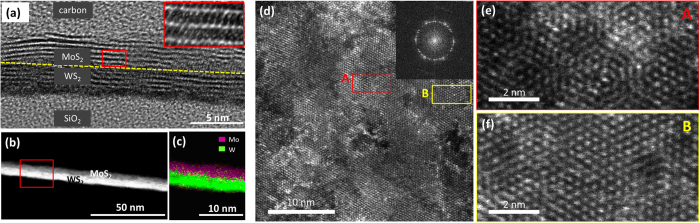
**(a)** Cross-sectional HRTEM of a vertically-stacked MoS_2_/WS_2_ heterostructure film, revealing a nearly clean heterointerface of MoS_2_/WS_2_. **(b)** Cross-sectional ADF-STEM image of a vertically-stacked MoS_2_/WS_2_ heterostructure, and **(c)** its corresponding EDS elemental mapping image. **(d)** Plane-view dark-field TEM image of a vertically-stacked MoS_2_/WS_2_ heterostructure film. **(e,f)** Close-up dark-field TEM images to show multiple Moiré fringes **(e)**: obtained from the red boxed region A in Figure (**d**). (****f****): obtained from the yellow boxed region B in Figure **(d)**.

The morphology of the stacked MoS_2_/WS_2_ films was further assessed by various TEM characterizations. Figure 3(a) shows a cross-sectional bright-field HRTEM micrograph of a stacked MoS_2_/WS_2_ film, revealing that the film consists of horizontally-grown 2D MoS_2_ and WS_2_ layers. Each material is indeed composed of ~4–5 layers, consistent with the results from the Raman and AFM characterizations. The inset shows the zoom-in image of the red boxed region showing the periodically-stacked 2D MoS_2_ in hexagonal (002) basal planes. Moreover, the image clearly reveals nearly atomically-sharp interfaces between MoS_2_ and WS_2_, further evidencing that the stacked MoS_2_/WS_2_ film well maintains the structural integrity of their constituting materials without alloying. The darker bright-field TEM imaging contrast from WS_2_ over MoS_2_ reflects that W is heavier than Mo. This observation is consistent with previous studies with mechanically stacked 2D TMDs^38^ and also confirms the ordered stacking of MoS_2_ and WS_2_ with seamless heterointerfaces. Further structural and chemical analysis of the MoS_2_/WS_2_ films was performed by scanning TEM (STEM) and energy dispersive x-ray spectroscopy (EDS) characterizations. Figure 3(b) shows an annular dark-field (ADF)-STEM image of a cross-sectioned MoS_2_/WS_2_ film different from the one in Fig. 3(a). The image clearly reveals a brighter image contrast for WS_2_ over MoS_2_ with a reversed image contrast in comparison to the bright-field TEM image, which is also consistent with previous observations with other in-plane 2D heterostructures^21^,^27^,^39^. The chemical compositions of vertically-stacked MoS_2_/WS_2_ films were characterized in EDS-STEM mode. Figure 3(c) shows the EDS-STEM elemental mapping image of the red boxed region in Fig. 3(b), revealing a highly localized spatial distribution of Mo and W on either side of the MoS_2_/WS_2_ stack. Figure 3(d–f) show plane-view dark-field TEM images of a vertically-stacked MoS_2_/WS_2_ film. Figure 3(d) shows that the MoS_2_/WS_2_ heterostructure material is a continuous film which possesses the poly-crystalline multiple layers composed of individual planar 2D grains. This poly-crystalline nature of the film is also confirmed by its corresponding fast Fourier transform (FFT) in the inset. Close-up inspections (Fig. 3(e,f)) further reveal multiple Moiré patterns with distinct fringe orientations at different locations (A,B in Fig. 3(d)) on the same sample. Figure 3(e) shows Moiré patterns resulting from stacks of multiple 2D layers whose basal planes are misaligned and rotated with respect to the [001] zone axis. This finding is qualitatively consistent with recent observations with vertically-stacked bilayer 2D TMD heterostructures integrated by manual exfoliations^15^. Meanwhile, Fig. 3(f) shows nearly single crystalline-like Moiré patterns which are attributed to that multiple 2D layers grew with aligned lattices on their basal planes. Such an epitaxial growth of MoS_2_/WS_2_ heterostructures was previously reported with materials grown by a co-evaporation CVD method^27^, which is attributed to that both MoS_2_ and WS_2_ possess identical lattice constants on their basal planes^40^. These characterizations together with the cross-sectional structural and chemical TEM analysis directly evidence that our CVD method produces 2D heterostructures composed of multiple MoS_2_ and WS_2_ layers in stacks with high spatial and chemical homogeneities.

**Figure 4:**
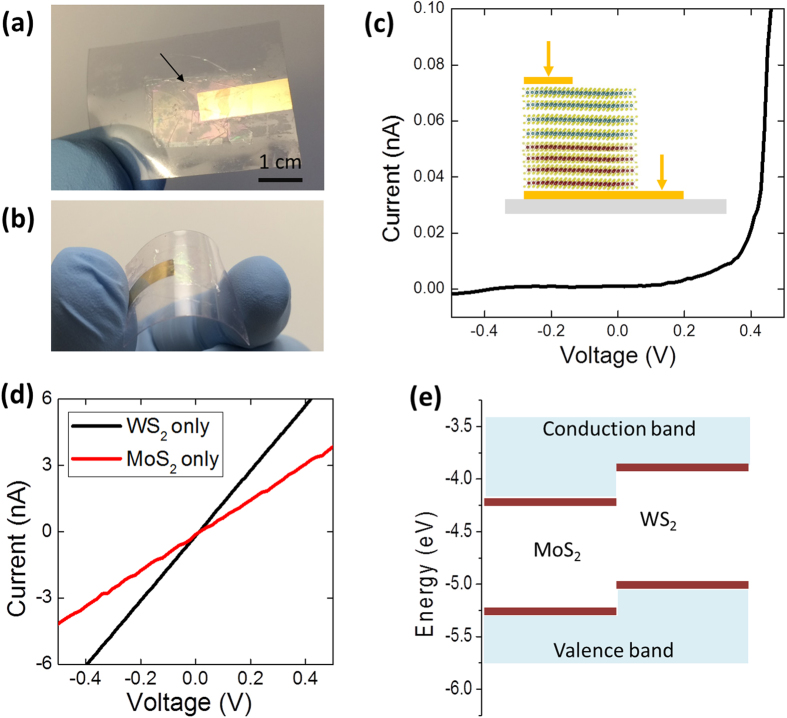
**(a)** Large area, vertically-stacked MoS_2_/WS_2_ heterostructure film transferred to a flexible insulating substrate with a pre-deposited electrode. The arrow indicates an area of the transferred film **(b)** The transferred film is under mechanical bending. **(c)** Two-terminal I–V measurement across a MoS_2_/WS_2_ heterointerface showing current rectification. **(d)** Two-terminal I–V measurements on individual MoS_2_ and WS_2_ films showing ohmic transports. **(e)** Energy band structure of a vertically-stacked MoS_2_/WS_2_ heterointerface.

In order to further assess the material quality of the vertical MoS_2_/WS_2_ heterostructure films, we performed electrical transport measurements across their heterointerfaces. One major advantage of these materials is their potential for novel device building blocks for large-area electronic devices compatible with unconventional substrates such as flexible polymers. To demonstrate this feasibility, we transferred the vertically-stacked MoS_2_/WS_2_ film onto a polyethylene terephthalate (PET) substrate by following a known polymethyl methacrylate (PMMA)-based transfer method^41^. Figure 4(a) shows an optical image of a large-area (~2 cm^2^) vertically-stacked MoS_2_/WS_2_ film transferred onto a PET substrate with a pre-deposited bottom electrode prior to the deposition of a top electrode. Figure 4(b) demonstrates the mechanical fleixlbity of the transferred film under bending. Two-terminal electrical characterizations were performed on the transferred film where metal contacts were separately made on MoS_2_ and WS_2_ as shown in the schematic in Fig. 4(c) inset. Figure 4(c) shows current-voltage (I–V) characteristics of a vertically-stacked MoS_2_/WS_2_ heterostructure film on a flexible PET substrate under a two-terminal room temperature transport measurement. A clear diode-like behavior with current ratio of >10^3^ at ±0.5 V is observed, which reflects current rectification across the hetorointerface. To confirm that the diode-like behavior was indeed originated from the heterointerface, two-terminal transport measurements were performed on MoS_2_-only and WS_2_-only films as shown in Fig. 4(d). Linear I–V characteristics reflecting ohmic transports were observed from the individual 2D films without heterointerfaces, which confirms that the rectification was resulted from the heterointerfaces. Figure 4(e) illustrates the ideal energy band structure of the MoS_2_/WS_2_ heterointerface, indicating a formation of type II heterojunction and built-in potentials which are responsible for the observed rectification^9^. Our results are also consistent with recent observations of recifications from vertically-stacked multilayer MoS_2_/WS_2_ heterostructure films demonstrated either by the manual integration^42^ or by the co-evaparition or sequential CVD methods^27^,^43^,^44^. It is worth pointing out that all these previous results were demonstrated with very small films (<10 μm^2^) unlike our present study.

## Conclusion

In summary, we report the centimeter-scale CVD growth of vertically-stacked few-layer 2D MoS_2_/WS_2_ heterostructure materials by sulfurizing stacks of Mo/W films. Comprehensive structural characterizations employing Raman spectrocscopy, AFM, and TEM/STEM evidenced that these novel materials are composed of 2D MoS_2_ and WS_2_ with well defined layer number, chemical homogeneity, and vdW heterointerfacs. Thorough elecrtrical characterizations revealed diode-like current rectification from these materials, further confirming that they preserve the electrical characteristics of individual constituent 2D components. The growth method presented in this study is intrinsically scalable and compatible with the existing complementary metal-oxide semiconductor (CMOS) process, suggesting high promise for developing novel electronic materials beyond contemporary Si technologies.

## Methods

### Growth Method

High quality Mo (thickness ~1–2 nm)/W (thickness ~1–2 nm) stacked films were fabricated by sequential magnetron sputtering of W and Mo targets (99.99% purity) on Si/SiO_2_ (300 nm SiO_2_) substrates at room temperature. Prior to the seed metal films deposition, Si substrates were thoroughly cleaned in acetone, isopropanol, and deionized water for 10 minutes, followed by drying using nitrogen gas. The as-deposited Mo/W films were then placed at the center of a LPCVD furnace equipped with a four inch quartz tube. Inside the tube, 100 mg of sulfur powder (99.5%, Sigma Aldrich) was placed in a separate alumina boat located at the upstream of the furnace. The tube was pumped down to 1 mTorr using a mechanical pump followed by heating up to 600 °C in 30 minutes with a held time of two hours. Throught the reaction, vaporized sulfur was carried by Ar gas to react with the metal films, which converts Mo/W films to MoS_2_/WS_2_ stacked films. Stacks of W/Mo films were also tested for the growth of vertically-stacked WS_2_/MoS_2_ films under the same growth conditions, which did not result in any noticeable difference compared to MoS_2_/WS_2_ films.

### Characterization

Thickness and surface morphology measurements of the grown films were performed by an AFM (Parks NX-10) system. Raman spectra of the films were collected in Almega XR Raman spectrometer equipped with an Olympus BX51 microscope, and spatial resolution of 1 μm with laser wavelength of 532 nm. Most of the structural and chemical analysis of the films were performed using a JEOL ARM200F FEG-TEM/STEM with a Cs-corrector for the electron probe. ADF STEM was performed with probe current of ~20 pA, condenser aperture of 30 μm, camera length of 8 cm, and collection inner angle of ~70 mrad. The scanning rate of the ADF images was employed with 6 μs per pixel and 512 × 512 pixels. STEM-EDS analysis was performed with EDAX detector (SDD type 80 T) and analysis software (AZtecTEM, Oxford). The cross-sectional TEM image in Fig. 3(a) was taken by TECNAI F20 S-Twin (FEI Co, Netherland) TEM. All TEM/STEM operations were conducted at an accelerating voltage of 200 kV. Cross-sectional TEM samples were prepared by focused ion beam (FIB)-based milling and lift-out techniques. As-grown MoS_2_/WS_2_ films were coated with a carbon film of ~100 nm thickness (108 C auto carbon coater, Cressington Scientific Instruments) and were subsequently cross-sectioned inside a FIB (Quanta 2D FEG, FEI). Ga ion milling (30 keV) was performed until the target area became electron transparent suitable for TEM imaging and the prepared specimen was transferred to a Cu TEM grid with a micromanipulator (Omniprobe) inside the FIB. For electrical measurements, as-grown MoS_2_/WS_2_ films on SiO_2_/Si substrates were first coated with PMMA thin films by spin-coating at 2500 rpm for 30 s, followed by baking at 130 °C for 30 min. The PMMA-covered substrates were then immersed in a buffered oxide etchant (BOE: aqueous NH4-HF solution), which led to the separation of the MoS_2_/WS_2_ stacked fims from the substrate etching away SiO_2_. The separted films were subseuently transferred to insulating substrates with pre-deposited bottom metal electrodes. Finally, PMMA was rinsed away with acetone and dionized water followed by the deposition of top electrodes on the MoS_2_/WS_2_ stacked fims. Gold (Au) was used for the electrode materials. Electrical measurements were performed at room temperature using an Agilent B2912A precision source/measure unit connected to a probe station with tungsten probes.

## Additional Information

**How to cite this article**: Choudhary, N. *et al.* Centimeter Scale Patterned Growth of Vertically Stacked Few Layer Only 2D MoS_2_/WS_2_ van der Waals Heterostructure. *Sci. Rep.* **6**, 25456; doi: 10.1038/srep25456 (2016).
